# Synchronizing the transcranial magnetic pulse with electroencephalographic recordings effectively reduces inter-trial variability of the pulse artefact

**DOI:** 10.1371/journal.pone.0185154

**Published:** 2017-09-21

**Authors:** Leo Tomasevic, Mitsuaki Takemi, Hartwig Roman Siebner

**Affiliations:** 1 Danish Research Centre for Magnetic Resonance, Centre for Functional and Diagnostic Imaging and Research, Copenhagen University Hospital Hvidovre, Hvidovre, Denmark; 2 Division of Physical and Health Education, Graduate School of Education, The University of Tokyo, Tokyo, Japan; 3 Department of Neurology, Copenhagen University Hospital Bispebjerg, Copenhagen, Denmark; University of Toronto, CANADA

## Abstract

**Background:**

Electroencephalography (EEG) can capture the cortical response evoked by transcranial magnetic stimulation (TMS). The TMS pulse provokes a large artefact, which obscures the cortical response in the first milliseconds after TMS. Removing this artefact remains a challenge.

**Methods:**

We delivered monophasic and biphasic TMS to a melon as head phantom and to four healthy participants and recorded the pulse artefact at 5 kHz with a TMS-compatible EEG system. Pulse delivery was either synchronized or non-synchronized to the clock of the EEG recording system. The effects of synchronization were tested at 10 and 20 kHz using the head phantom. We also tested the effect of a soft sheet placed between the stimulation coil and recording electrodes in both human and melon.

**Results & conclusion:**

Synchronizing TMS and data acquisition markedly reduced trial-to-trial variability of the pulse artefact in recordings from the phantom or from the scalp. Reduced trial-to-trial variability was also observed at high sampling frequencies. The use of a soft sheet reduced the variability in recordings on the head phantom, but not in human participants. Effective reduction of the trial-to-trial variability renders it possible to create an artefact template for off-line filtering. Template-based subtraction of the artefact from the EEG signals is a prerequisite to effectively recover the immediate physiological response in the stimulated cortex and inter-connected areas.

## Introduction

Measuring electroencephalographic (EEG) responses to transcranial magnetic stimulation (TMS) provides unique possibilities to study the physiological response of the human cortex in-vivo. This is particularly relevant for cortical areas that do not provide other measurable responses to TMS, such as the motor evoked potential in the motor cortex or the perception of phosphenes in the visual cortex [1,2]. However, cortical physiological responses to TMS are covered by stimulation-induced artefacts in the EEG signals, which can be of physiological and instrumental origin [3]. The artefact that affects the EEG signal the most is caused by the TMS pulse, provoking a strong electric perturbation of the recorded signals [4]. The standard procedure to clean this artefact is to remove the EEG interval during which the artefact is present and to interpolate the missing trace [5]. This procedure leads to a definitive loss of the signals in the period of the pulse artefact, rejecting the very early physiological cortical response 5–6 ms after the TMS pulse.

Alternatively, one may recover the physiological cortical response in the first few milliseconds following the TMS pulse by reducing the pulse artefact. This can be achieved by reducing its variability and creating a reliable artefact template. The template can be applied for pattern-matched off-line filtering, which has been successfully used to unveil the cortical activity when combining EEG and functional MRI. This artefact cleaning method subtracts a template of the magnetic field gradient from the noisy EEG signals of which the amplitude is comparable to the TMS pulse artefact [4,6]. The present study was, therefore, designed to identify the proper experimental setting to reduce the variability of the TMS pulse artefact in signals recorded by the EEG system. First, we tested the impact of the synchronization between the TMS pulse delivery and the signal acquisition clock of the EEG system on the artefact variability. The temporal dynamics of the TMS pulse contains much higher frequency components than the standard EEG sampling frequency [4]. Thus, the shape of subsequent artefacts will vary considerably between trials due to temporal aliasing, if the stimulus is randomly delivered within a sampling interval of the recording system [7]. Second, we examined whether placing a soft sheet between the TMS coil and the EEG electrodes could further decrease the variability of the pulse artefact.

## Methods

**Fig 1:**
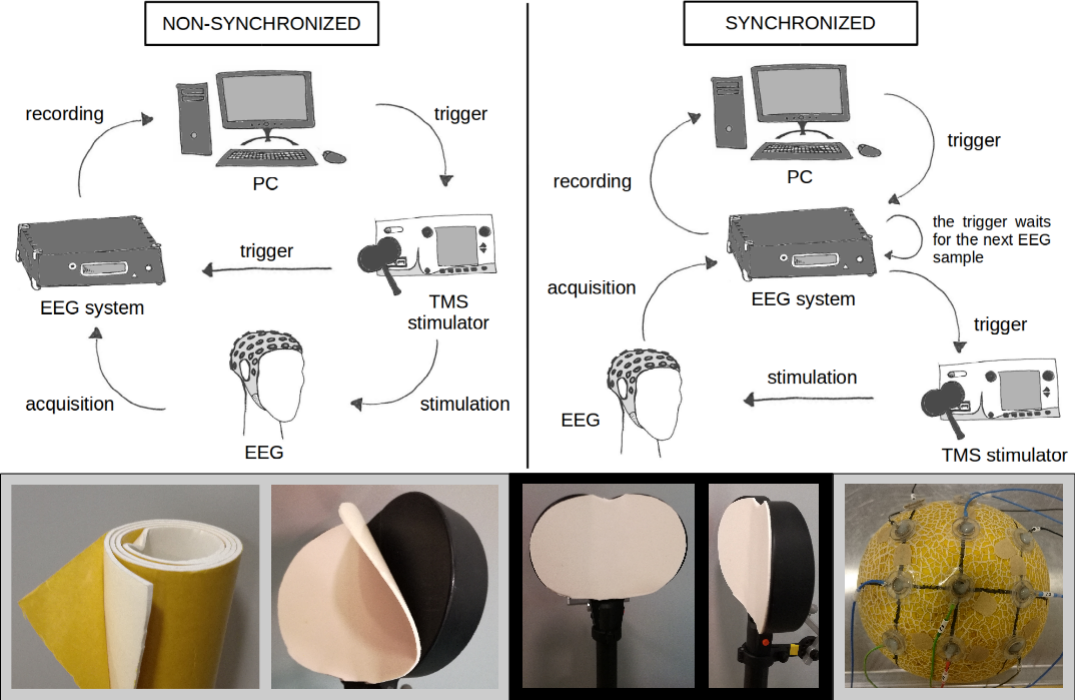
Experimental framework. Upper panel: The schematic representations of two arrangements: non-synchronized (left) and synchronized (right). In the synchronized version the EEG system is controlling the timing of the stimulation by linking it to the timing of the EEG sampling occurrence. Lower panel: The three photos on the left side show the used auto-adhesive soft silicone rubber sheet and how the sheet was attached to the lower coil surface. The photo on the right side shows the arrangement of the electrodes on the melon.

We performed our experiments on a head phantom as well as in four human volunteers. We used a melon (*Cucumis melo*) as head phantom to avoid any physiological confounds on the variation of the artefacts (Fig 1, right bottom). The same procedure has been already used for the same purpose, because *Cucumis melo* has similar dielectric properties to the human skin [8,9,10]. In addition, four healthy participants without history of neurological injury or disease or any other contraindication to TMS [11] were enrolled. All the participants gave their written consent to participate in the study. Experimental procedures in human volunteers had been approved by the Ethics Committee of the Capital Region of Denmark (H15008824).

For each condition, 100 magnetic stimuli were generated using a MagPro R100 stimulator and delivered at 60% of the maximal stimulator output through a MC-B70 figure-of-eight coil (MagVenture, Farum, Denmark) with 1.5±0.9 s inter-stimulus interval. The coil was placed over the central electrode in both phantom and human experiments. Nine EEG Ag/AgCl electrodes were placed in a 3×3 grid with 3 cm distance between the electrodes on the head phantom. In human participants, a M10 equidistant electrode cap with 63 TMS compatible Ag/AgCl electrodes was used (EasyCap GmbH, Herrsching, Germany). The impedances were monitored throughout the experiment with the optimal range being set <5 kΩ. Data were acquired with a NeurOne Tesla EEG amplifier (Mega Electronics, Kuopio, Finland) at 5 kHz sampling rate with 1250 Hz low-pass filter and DC filter. The operating range was ±430 mV and sensitivity was 51 nV/bit.

We tested the variability of the magnetic pulse artefact using monophasic and biphasic stimulation in three conditions. In the first condition, the pulse delivery was not coupled with the clock of the EEG system (i.e., non-synchronized condition). In the second condition, the delivery of the TMS pulse was synchronized to the EEG signal acquisition clock (i.e., synchronized condition); the experimental set-up is shown in Fig 1. In the third condition, the pulse was synchronized to the clock and we placed a soft silicone rubber sheet (1.5 mm thickness; Fig 1, lower panel) between the stimulation coil and the EEG electrodes (i.e., synchronized with sheet condition). The synchronization was achieved by sending the trigger to the EEG system, which then waited for the next EEG sample before sending the trigger to the stimulator (Fig 1, upper panel). This option is not only available in the EEG system employed in this study but also implemented in other commercial EEG systems, for instance the SyncBox made for Brain Products EEG systems. In addition, the synchronized and non-synchronized conditions were tested at different sampling rates (5, 10 and 20 kHz) on the head phantom with a low-pass filter of 1250, 1500 and 5000 Hz respectively. This is to test whether synchronization shows a decrease in the artefact variability also at higher sampling rates, where differences in the temporal distance between the EEG sample and the magnetic pulse across trials, which provokes the variability of the artefact, are smaller.

Data analyses involved trials time-locked to the pulse artefact for each channel and condition. The variability of the pulse artefact was evaluated by subtracting the average of the trials from single trial values and normalizing the result with the maximum peak-to-peak amplitude of the average. From the obtained values, those corresponding to the time point with maximal variance among trials were extracted for statistical analysis. It should be noted that the time point was different across channels and conditions. To assess differences of the artefact variability between conditions, we performed analysis of variance with aligned rank transform (ART-ANOVA) [12] in two datasets. One consisted of data acquired in the non-synchronized and synchronized conditions without sheet, and the other consisted of data acquired with and without sheet while TMS and EEG systems were synchronized. For the phantom data, we performed ART-ANOVA with factors of channel positions (ch1−9), sampling rates (5, 10, 20 kHz), and conditions (synchronized or non-synchronized) on the first dataset and with factors of channel positions and conditions (with or without a sheet) on the second dataset. For human data, we performed ART-ANOVA with factors of channel positions (ch1−63) and conditions on both datasets. Statistical tests on data obtained from humans were separately performed in each individual to stress the reproducibility of the procedure. If the ANOVA yielded a significant p value, post-hoc pairwise comparisons were performed using Wilcoxon rank-sum test with Bonferroni-Holm correction. The type I error was set to 5%.

## Results

### Phantom: Effects of the synchronization and the sampling rate on the artefact variability

**Fig 2:**
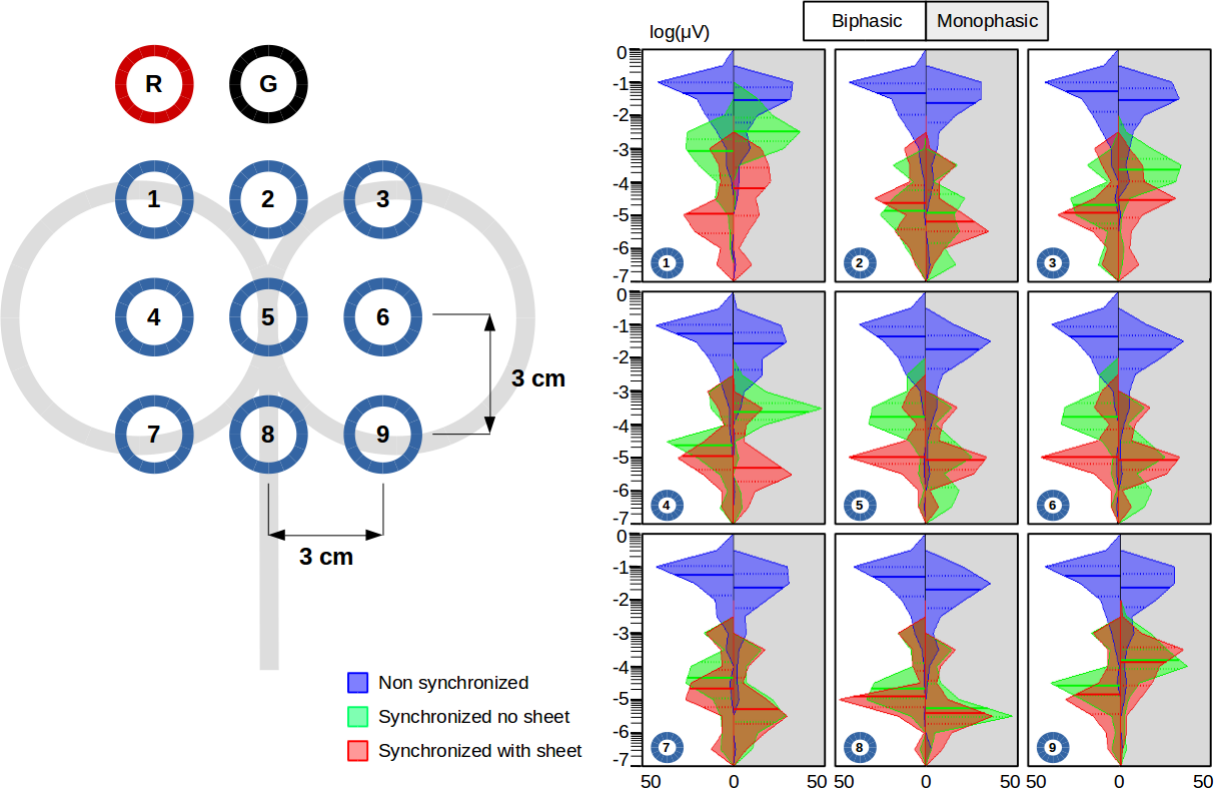
Electrodes' placement and estimation of variability in the head phantom. Left panel: The grid of the electrodes as they have been placed on the head phantom. The centre of the magnetic coil was located over the electrode number 5. Inter-electrode distance was 3 cm. The locations of the reference and the ground electrodes were shown as R and G, respectively. Right panel: Distribution of the variability of the pulse artefacts for each channel. Index of the variability is absolute difference between the amplitude at each trial and the averaged amplitude over all trials. The number shown at the left bottom of each box represents the electrode number. The left side of each box represents the results of the biphasic pulse and the right side (shaded by grey colour) represents the monophasic pulse results. The full line in the plots is the median. The dashed lines are the 25 and 75 percentiles.

The effect of the synchronization markedly reduced inter-trial variability of the artefact caused by the monophasic and biphasic pulse. The reduction in variability was prominent across all 9 channels (Fig 2 right, between blue and green). The ART-ANOVA performed on the phantom data in non-synchronized and synchronized conditions revealed significant main effects of the factors conditions (biphasic: F_1,5346_ = 6552, p<0.001; monophasic: F_1,5346_ = 5922, p<0.001), channel positions (biphasic: F_8,5346_ = 2.56, p = 0.009; monophasic: F_8,5346_ = 21.4, p<0.001), and sampling rates (biphasic: F_2,5346_ = 184, p<0.001; monophasic: F_2,5346_ = 114, p<0.001). Moreover, a significant interaction among the synchronization, channel positions, and sampling rates was found (biphasic: F_16,5346_ = 9.94, p<0.001; monophasic: F_16,5346_ = 6.51, p<0.001). Post-hoc comparisons revealed that the variability of the pulse artefact was larger for the non-synchronized than the synchronized conditions regardless of the channel locations and the sampling rates (p<0.001). In the non-synchronized condition the artefact variability was smaller for the 20 kHz sampling than the 5 and 10 kHz in the 8 out of 9 channels irrespective of the pulse shape (p<0.05) and no statistically significant difference was found between the 5 and 10 kHz. We also found significant differences of the artefact variability among different sampling rates in the synchronized condition, but this was not consistent among channels and sampling rates, suggesting that higher sampling rate reduces artefact variability only in the non-synchronized condition.

### Phantom: Effects of a sheet between the coil and the EEG electrodes on the artefact variability

Placing a soft sheet between the coil and the electrodes further reduced pulse variability (Fig 2 right, between green and red). The ANOVA assessing the data acquired with and without the sheet, while TMS and EEG systems were synchronized, revealed significant main effects of the sheet (biphasic: F_1,1782_ = 210, p<0.001; monophasic: F_1,1782_ = 288, p<0.001) and channel positions (biphasic: F_8,1782_ = 22.4, p<0.001; monophasic: F_1,1782_ = 148, p<0.001), and a significant interaction among sheet and channel positions (biphasic: F_8,1782_ = 31.4, p<0.001; monophasic: F_1,1782_ = 64.6, p<0.001). Post-hoc comparisons revealed that the variability of the pulse artefact was smaller for channels 1 and 6 when a sheet was placed between coil and electrodes, regardless of the pulse shape (p<0.05). In addition, the sheet significantly reduced the variability of the monophasic artefact at the channel 3 and 4 and the biphasic artefact at the channel 5 (p<0.05). No significant increase of the artefact variability by placing a sheet was observed for any channel and pulse shape.

### Scalp recordings: Effects of the synchronization on the artefact variability

**Fig 3:**
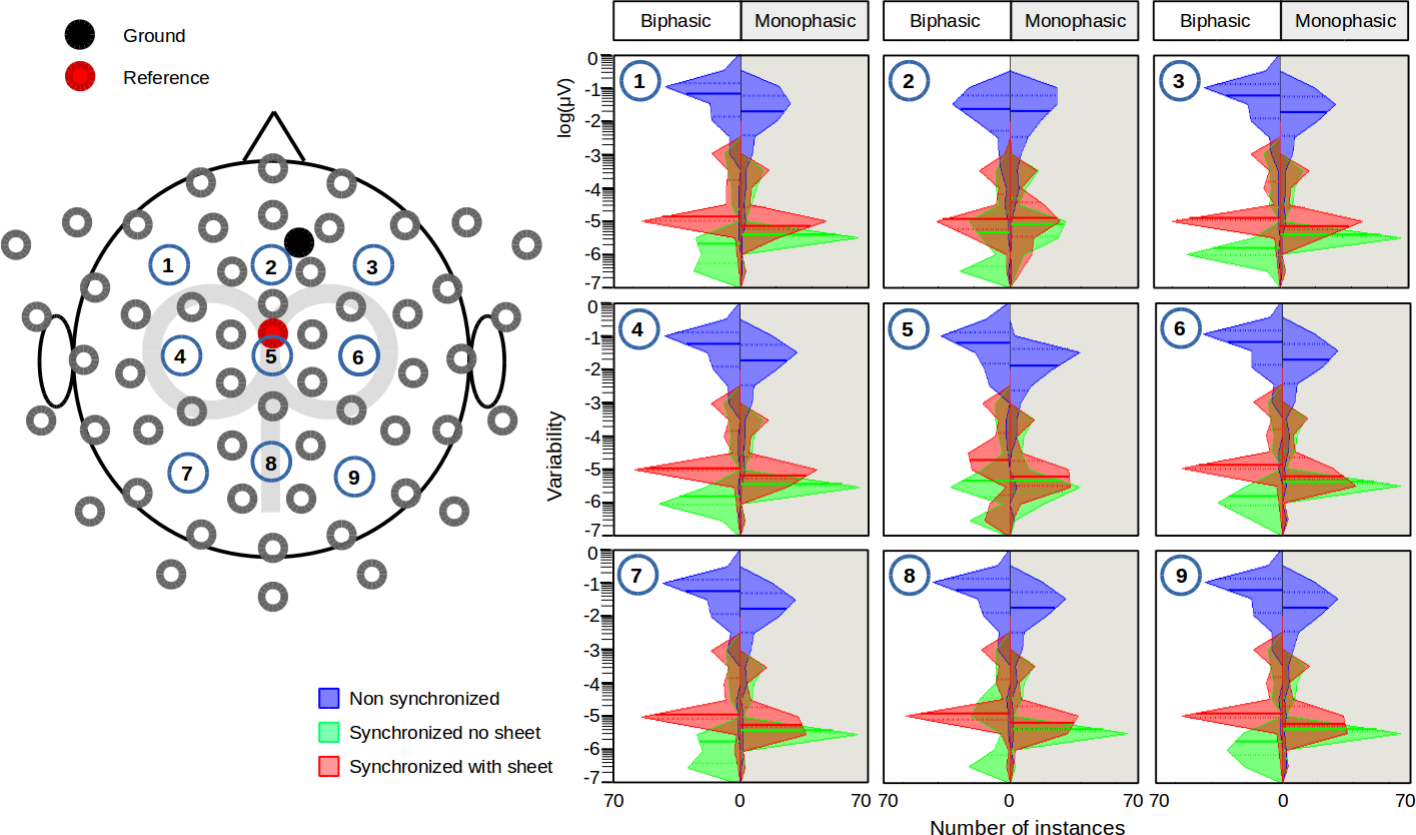
Electrodes' placement and estimation of variability in an explicative human participant. Left panel: The placement of the electrodes on the EEG cap. The centre of the magnetic coil was located over the electrode number 1. The locations of the reference and the ground electrodes were shown as R and G, respectively.Right panel: Distribution of trial-to-trial variability of the pulse artefacts for selected channels. Those EEG channels were chosen for which the position relative to the coil was similar to the one in the head phantom experiment. The absolute difference between the amplitude at each trial and the averaged amplitude over all trials was taken as index of trial-to-trial variability. The number shown at the left bottom of each box represents the electrode number. The left side of each box represents the results of the biphasic pulse and the right side (shaded by grey colour) represents the monophasic pulse results. The full line in the plots is the median. The dashed lines are the 25 and 75 percentiles.

**Fig 4:**
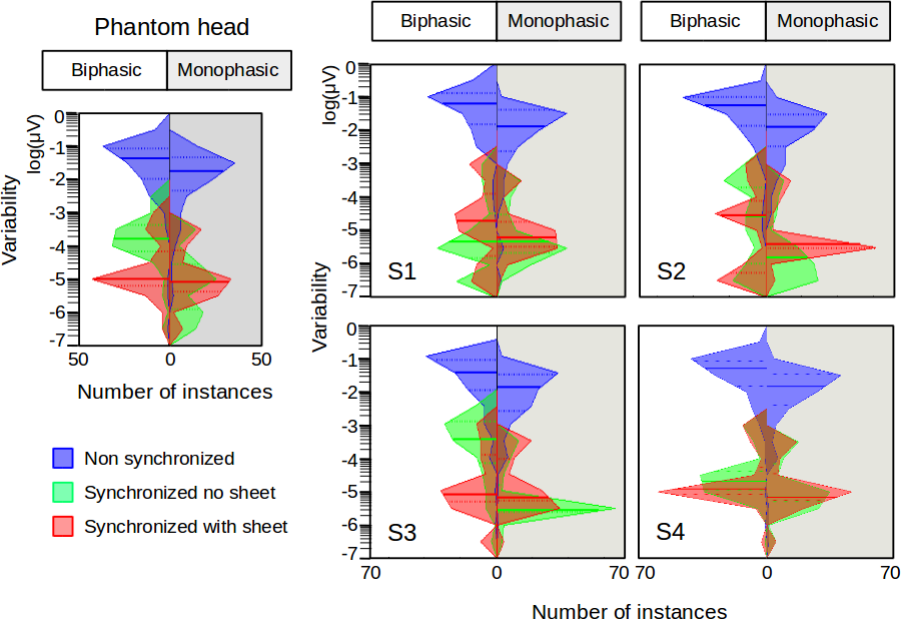
Comparison of the effects. The channel under the coil for the head phantom and human subjects has been chosen to show the effects of the synchronization between the TMS and EEG recordings, both with biphasic and monophasic pulse shape.

Confirming the results obtained with the head phantom, the synchronization of the TMS pulse delivery and the EEG signal acquisition clock drastically reduced inter-trial variability of the artefact present in human EEG signals. Representative data from a single participant are shown in Fig 3. In this participant, a significant interaction was found between conditions and channel positions (biphasic: F_62,12474_ = 12.4, p<0.001; monophasic: F_62,12474_ = 4.12, p<0.001). The reduction in variability was statistically significant for all channels with both the biphasic and the monophasic pulse (Fig 3 right, between blue and green), but the effect size was largely different among the channel positions. The ANOVAs separately performed on each individual data set in non-synchronized and synchronized conditions revealed a significant main effect of the factor conditions in all participants (biphasic: F_1,12474_ = 2051−2735, p<0.001; monophasic: F_1,12474_ = 1890−3122, p<0.001). A significant main effect of the factor channel positions (biphasic: F_62,12474_ = 2.76−10.7, p<0.001; monophasic: F_62,12474_ = 2.42−4.38, p<0.001) and a significant interaction of the conditions and channel positions (biphasic: F_62,12474_ = 2.29−12.4, p<0.001; monophasic: F_62,12474_ = 2.07−4.12, p<0.001) was also found in all participants. Post-hoc comparisons revealed that the variability of the pulse artefact was larger for the non-synchronized than the synchronized conditions regardless of the channel locations and the pulse shapes in all participants, but the level of significance varied among the channel locations in all participants for both pulse shapes (biphasic: p = 2.2×10^−23^−2.4×10^−31^, monophasic: p = 1.0×10^−25^−3.6×10^−32^; Fig 4). In addition, the artefact variability was statistically different among several channels in the both conditions (p<0.05), but the pairs of channels showing the statistical difference were not consistent across participants. Significant differences were found in 88−93% (with monophasic) and 69−82% (with biphasic) of the total number of channel pairs in the non-synchronized condition. This number decreased to 22−71% (with monophasic) and 20−60% (with biphasic) in the synchronized condition.

### Scalp recordings: Effects of a sheet between coil and electrodes on artefact variability

In contrast to recordings in the head phantom, placing a soft sheet between the coil and the EEG electrodes did not consistently help to reduce the artefact variability in electrophysiological recordings from the scalp. The ANOVAs assessing the data acquired with and without the sheet, while TMS and EEG systems were synchronized, revealed significant main effects of the sheet (biphasic: F_1,12474_ = 5.23−1357, p = 0.02− p<0.001; monophasic: F_1,12474_ = 207−2222, p<0.001) and channel positions (biphasic: F_62,12474_ = 2.59−16.6, p<0.001; monophasic: F_62,12474_ = 1.44−6.61, p<0.001) in all participants. The sheet effectively reduced artefact variability for biphasic stimulation in subjects 2, 3 and 4, but increased variability for the biphasic stimulation in subject 1 and for monophasic stimulation in all participants (Fig 4 right, between red and green). A significant interaction among sheet and channel positions was found with the biphasic stimulation (F_62,12474_ = 2.86−18.3, p<0.001) but not with the monophasic stimulation (F_62,12474_ = 0.31−1.00, p = 0.48−1.00).

## Discussion

**Fig 5:**
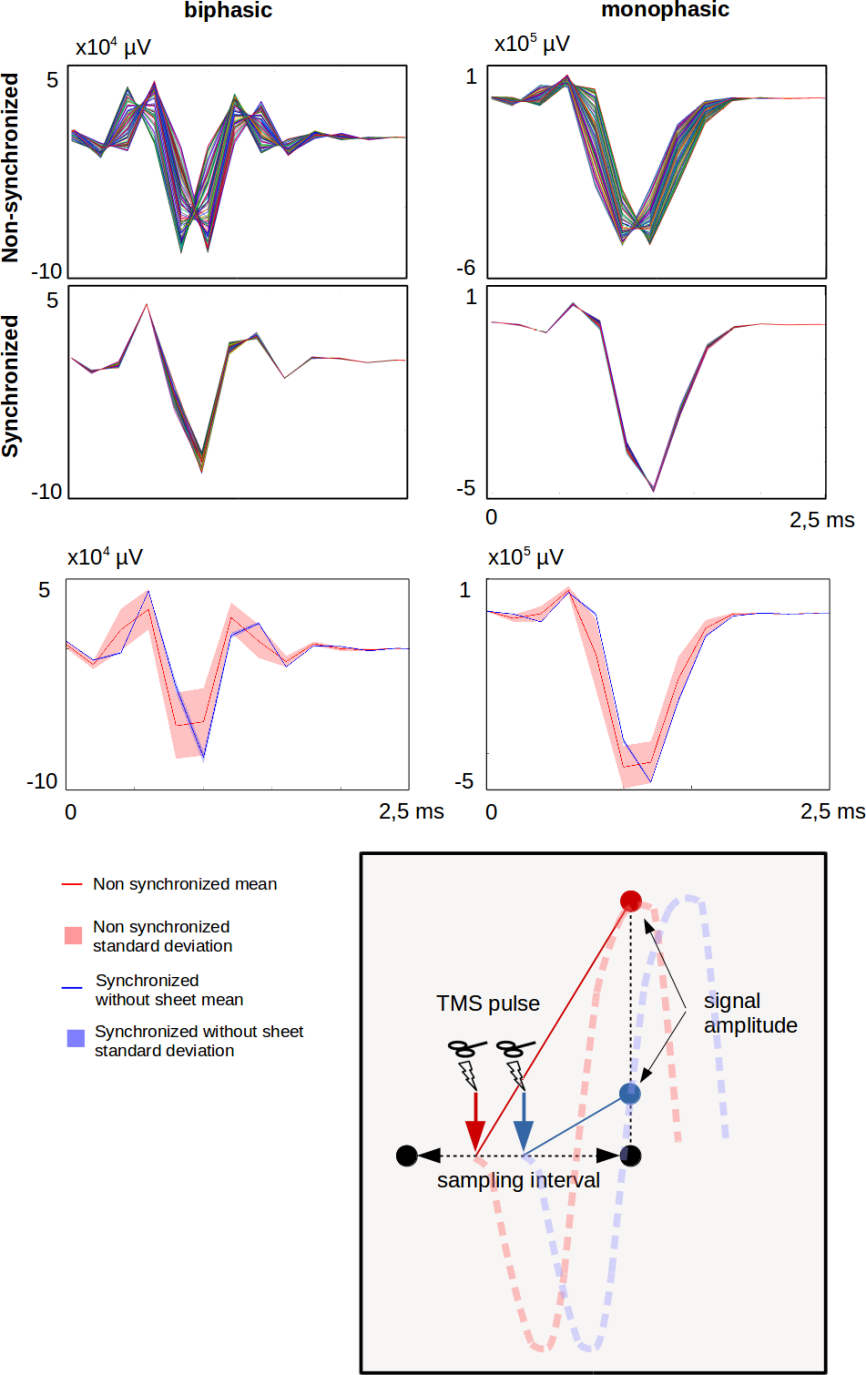
Effects of synchronization. Upper panel: The first two rows show an overlay of 100 traces of the TMS pulse artefact (left column: biphasic pulse, right column: monophasic pulse). There is a large difference in inter-trial variability between the non-synchronized (first row) and non-synchronized (second row) recording condition, showing a marked reduction in variability when the TMS pulse was synchronized with the onset of EEG sampling. The third row shows the mean (line) and the standard deviation (shadow) of the pulse artefact that is superimposed for the non-synchronized (red) or synchronized (blue) conditions. Lower panel: Schematic illustration of how the pulse artefact varies in the recorded signal in case of under-sampling. TMS pulse falling in two different time points within a sampling interval (blue and red arrows in the schema) will be measured with different amplitudes at the first data sampling point following the TMS pulse. The pulse artefacts in the non-synchronized condition become strongly variable as shown by the different signal amplitudes.

We showed that the experimental setting strongly influence trial-to-trial variability of the pulse artefact in TMS-EEG experiments. Confirming our hypothesis, the lack of synchronized timing of TMS pulse delivery and EEG signal sampling contributes to the trial-to-trial variability (Fig 5, upper panel). The dynamics of the pulse artefact has much higher frequency components than the EEG sampling rate in standard TMS/EEG experiments [4]. If the pulse event occurs in a random time point between two EEG samples, the first EEG sample after TMS will capture the pulse artefact at different positions of its dynamics, resulting in large variability in the amplitude of the pulse artefact (Fig 5, lower panel). By synchronizing the pulse delivery with the EEG system clock, the dynamics of the artefact are captured always at the same time and amplitude position, decreasing drastically the variability of the recorded artefact. This effect was still present at higher sampling frequencies, where the temporal distance between two EEG samples is smaller, suggesting that the drastic reduction of the artefact variability shown in this study can be achieved only by synchronizing the pulse delivery with the EEG system clock.

We also tested whether placing a thin silicon rubber sheet between the coil and the electrodes can additionally reduce the variability of the pulse artefact, and we obtained inconsistent results between a head phantom and human participants. The sheet helped to reduce the variability in a head phantom, while effects were inconsistent for recordings from human scalp. Other possible causes for the inter-trial variability of the artefacts include changes of the position of the coil with respect to the electrodes during the experiment, changes of the electrode impedance, and heating of the electrodes due to repetitive stimulation. The impact of these factors on trial-to-trial variability remains to be addressed in future studies.

The present study provided the first empirical evidence that synchronization of TMS delivery and the clock controlling EEG recordings effectively reduces inter-trial variability of the pulse artefact in TMS-EEG experiments. This reduction of the artefact variability greatly facilitates the creation of an artefact template, which can be subtracted from the noisy EEG signals to recover the physiological cortical responses immediately after the administration of TMS. This will be crucial for investigating early brain reactivity during the first few milliseconds after the stimulation, such as GABAergic inhibition within the stimulated brain area [13]. This inhibition has been shown so far with the paired pulse stimulation-evoked electromyogram, but to the best of our knowledge, corresponding EEG fluctuations occurring right after the first conditioning pulse remain to be shown. However, more studies are needed to fully recover the early cortical brain response during the first milliseconds after delivery of the TMS pulse. Please note that this does not only require to clean the stimulus artefact, but also the muscular artefact which is provoked by a TMS-evoked muscle twitch or caused by direct axonal stimulation of the peripheral nerves supplying the cranial muscles [9]. The muscle artefact is of physiological origin and shows different timing and shape. Hence, it is unrelated to hardware synchronization and has not been investigated further in this study.

## Supporting information

S1 File Influence of syncronization on the artefact variability (head phantom). The data are composed by 3-dimentional matrices with 100 trials and 9 channels (trials X time X channel).
(ZIP)

S2 File Sampling frequency rate impact on the synchronization (head phantom). The data are composed by 3-dimentional matrices with 100 trials and 9 channels (trials X time X channel).
(ZIP)

S3 File Syncronization and artefact variability in 4 human subjects. The data are composed by 3-dimentional matrices with 100 trials and 63 channels (trials X time X channel).
(ZIP)
